# Hospital Questions Which Press

**Published:** 1903-02-07

**Authors:** 


### HOSPITAL QUESTIONS WHICH PRESS.

Introductory.

A critical stage has been reached in the history of our voluntary hospital system in London. Owing no doubt to the enthusiasm and energy which have been displayed in upholding and expanding the cause of medical charities by several influential people, more especially during the last few years, the public is learning to take a deeper and more personal interest in, and a more active attitude towards, the London hospitals. It may well be that having contributed more largely towards the support of these institutions, the general public feels that by so much the more has it a right to make its voice heard whenever there is an important hospital problem under consideration. A study of the Press is sufficient to demonstrate that a very much more acute general interest is being displayed towards all hospital questions. To no one will this increasing notice in the newspapers be more welcome than to those who are responsible for the management of the hospitals, even although they may feel that some of the criticisms passed upon their actions may be unjust or based upon insufficient knowledge. It at least signifies without doubt an improved desire on the part of the public to become well acquainted with the working and administration of these charities. Let us hope, too, that people will take a more active part in advancing their welfare. By creating public discussion the Press is doing a great service in helping to still further extend the area of public interest in the hospitals.

